# Fermentation Preparation of Umami Sauce and Peptides from Kelp Scraps by Natural Microbial Flora

**DOI:** 10.3390/foods14101751

**Published:** 2025-05-15

**Authors:** Jizi Huang, Ruimei Wu, Yijing Wu, Feiyang Liang, Yiming Chen, Fujia Yang, Huawei Zheng, Zonghua Wang, Huibin Xu, Songbiao Chen, Guangshan Yao

**Affiliations:** 1 Fuzhou Institute of Oceanography, Minjiang University, Fuzhou 350108, China; 18289864698@163.com (J.H.); yijingwu@mju.edu.cn (Y.W.); yuyuyuyu03@163.com (F.L.); chenyiming@stu.mju.edu.cn (Y.C.); yangfujia@mju.edu.cn (F.Y.); zhw@mju.edu.cn (H.Z.); wangzh@fafu.edu.cn (Z.W.); sbchen@fjage.org (S.C.); 2 School of Future Technology, Fujian Agriculture and Forestry University, Fuzhou 350007, China; 3 Jinshan College, Fujian Agriculture and Forestry University, Fuzhou 350007, China; ruimeiwu@126.com; 4 Key Laboratory of Cultivation and High-Value Utilization of Marine Organisms in Fujian Province, Xiamen 361000, China

**Keywords:** kelp sauce, natural microbial flora, fermentation, alginate lyase, protease, umami peptides, T1R1–T1R3

## Abstract

Kelp (*Laminaria japonica*) is renowned for its rich content of flavor-enhancing amino acids and nucleotides; however, approximately 40% of kelp, including the thin edges and root areas, is discarded during its processing due to its inferior taste. To recycle these kelp byproducts, we have cultivated a functional microbial consortium through continuous enrichment. Analysis via 16S rRNA sequencing has shown that during the three fed-batch fermentation stages of kelp waste, the microbial community was predominantly and consistently composed of three phyla: *Halanaerobiaeota*, *Bacteroidota*, and *Proteobacteria*. At the genus level, *Halanaerobium* emerged as the dominant player, exhibiting a trend of initial increase followed by a decline throughout the fermentation process. Enzymes such as alginate lyases and both acidic and neutral proteases were found to play crucial roles in the degradation of kelp residues into sauces. Notably, electronic tongue analysis revealed that the fermented kelp sauce demonstrated strong umami characteristics. Furthermore, four novel umami peptides, EIL, STEV, GEEE, and SMEAVEA, from kelp were identified for the first time, with their umami effect largely attributed to strong hydrogen bond interactions with the T1R1–T1R3 umami receptors. In conclusion, this study proposed a sustainable method for kelp by-product utilization, with implications for other seaweed processing.

## 1. Introduction

As a representative species of kelp, the brown alga *Laminaria japonica* (commonly designated as “kombu” in Japan) has constituted a fundamental marine medicinal food resource for centuries [1,2]. This perennial seaweed, taxonomically classified as the *Laminaiales* order and *Laminariaceae* family, exhibits particular cultural significance in East Asian culinary traditions. In China, where it is systematically referred to as Hai-Dai, this macroalga has maintained dual culinary and therapeutic applications for over two millennia, with historical records documenting its systematic cultivation and pharmacological utilization since the Han Dynasty [3,4]. This species is renowned for its more than 40 bioactive compounds and is widely regarded as one of the healthiest seafoods available. Research indicates that polysaccharides and proteins constitute approximately 80% of their dry weight while they are also rich in carotenoids, polyphenols, vitamins, and essential minerals like iodine, magnesium, calcium, iron, and zinc [2,5]. As a brown macroalga of ecological and economic significance, *L. japonica* is extensively distributed and cultivated on a large scale across Northern Asia, including China, Japan, and Korea. According to the Food and Agricultural Organization (FAO), it holds the title of the world’s highest-yielding seaweed, with an annual production reaching up to 4.8 million tons [6]. The majority of harvested kelp is consumed fresh, salted with sea salt, or freeze-dried as a nutritious daily food [5]. However, during food processing, about 40% of the total kelp output, including the thin edges and root parts, is discarded due to their inferior taste. This raises the question of how best to utilize these kelp byproducts effectively. Addressing this issue could lead to enhanced sustainability and value addition within the seaweed industry.

As a traditional Chinese herbal medicine, kelp has been utilized for more than a thousand years due to its anti-diabetic, anti-obesity, and anti-tumor properties [2,7]. However, much of the research aimed at developing medicine, functional foods, and raw food materials from kelp has predominantly focused on its polysaccharide content [7,8], with relatively little attention given to its protein composition. Recently, there has been growing interest in peptides derived from kelp proteins, which have demonstrated a range of bioactivities. For example, kelp-derived peptides have been shown to suppress liver cancer in vitro by inducing apoptosis, potentially through multiple signaling pathways [9]. A cyclic tetrapeptide named LCP-3, isolated from kelp, exhibits anti-colorectal carcinoma effects by promoting apoptosis both in vitro and in vivo [1]. Additionally, two peptides obtained from the protein hydrolysate of *Laminaria digitate* have been found to possess Angiotensin-1-Converting Enzyme (ACE-1) inhibitory activities [10].

To fully exploit and enhance the resource value of kelp, various extraction, separation, and transformation techniques are employed for its intensive processing, which can be categorized into chemical, zymolytic, and microbial fermentation methods [11]. Multiple organic solvents were previously used to extract and prepare polysaccharides and peptides from brown seaweed [12]. For example, alginate polysaccharide and fucoidan are extracted from kelp biomass by using H_2_O_2_ or KOH, and these polysaccharides can be further depolymerized into oligosaccharides by acid or base [13]. Alkaline treatment is used to extract seaweed proteins for further bioactive peptide preparation [14]. Generally, these above chemical methods have several drawbacks: strong acids and alkalis can easily destroy the unstable and nutritious components in kelp, residual organic chemicals bring food safety concerns, and they are environmentally unfriendly [12,14]. Although the biotransformation of kelp by multiple enzymes into valued oligosaccharides or bioactive peptides is feasible, the low catalytic efficiency for some enzymes leads to a higher processing cost [15]. In comparison, microbial fermentation is a highly promising method for converting brown algae into bioactive peptides and oligosaccharides. Firstly, microbial fermentation transforms these indigestible cell wall components into absorbable and nutritional ingredients, such as oligosaccharide and peptides. Secondly, during fermentation, microbes produce a large number of primary or secondary metabolites with unique biological activities, enhancing the nutritional value of seaweeds. It has been reported that the fermentation of *Pyropia yezoensis* by a combination of several microbes increased pleasant fragrances, such as alcohols, ketones, and esters [16]. Last but not least, toxic and hazardous compounds are eliminated after microbial fermentation [17].

The taste of umami has been widely recognized as the fifth basic taste, identified after sweet, sour, salty, and bitter. Umami has emerged as a crucial factor influencing food quality, with the enhancement of the content of umami compounds to improve overall palatability becoming an important trend in the food industry. Ingredients such as monosodium glutamate (MSG), nucleotides, and umami peptides are known to impart this savory flavor [18]. In 1907, Kikunae Ikeda first isolated and identified MSG as the umami component in kelp, marking the beginning of umami’s recognition [19]. However, while kelp contains glutamate, its concentration alone is not sufficiently high to justify the industrial production of L-glutamine from this source; hence, wheat gluten is predominantly used for this purpose. It is reasonable to speculate that other components in kelp, alongside glutamate, collectively contribute to its characteristic umami taste. Despite this, the full spectrum of these umami-contributing substances within kelp remains largely unexplored.

In this study, we transformed kelp processing by-products into a flavorful kelp sauce through microbial fermentation. The composition of the functional microbial community involved in this process was characterized using 16S rRNA sequencing at three distinct stages of fermentation. Additionally, we analyzed the variation in active enzymes responsible for depolymerizing polysaccharides and proteins throughout the fermentation process. A LC–MS analysis identified four major peptides that contribute to the umami taste of the kelp sauce. Electronic tongue analysis was employed to assess the taste profiles of the kelp sauce and the isolated umami peptides. In addition, we predicted the structure model of umami receptor TAS1R1–TAS1R3 and performed molecular docking studies to elucidate the interaction mechanisms between the receptor and the umami peptides. In conclusion, new umami peptides were first identified from seaweed kelp, providing a route for the development of discarded kelp byproducts.

## 2. Materials and Methods

### 2.1. Materials and Chemicals

The kelp byproducts were supplied by TianYuan Shui Chan of Fujian Co. (Fuzhou, China). Polymannuronic acid (PolyM), polyguluronic acid (PloyG), and alginate derivatives were purchased from Qingdao BZ Oligo Biotech (Qingdao, China). The synthesis of four custom peptides, including EIL, GEEE, STEV, and SMEEVEA, was undertaken by DGpeptides Co. (Wuhan, China). High-fidelity Pfu DNA polymerase was purchased from TransGen Biotech (Beijing, China). The hammarsten-grade casein was purchased from Sigma-Aldrich (Saint Louis, MO, USA). All other chemical reagents were supplied by Sangon Biotech Co., Ltd. (Shanghai, China).

### 2.2. Microbial Fermentation

These two yeast strains, including *Saccharomyces cerevisiae* mj1003 (isolated from rice wine) and *Pichia pastoris* X33 [20], were inoculated into YPD medium (peptone 20 g/L, glucose 20 g/L, and yeast extract 10 g/L) to culture at 30 °C until the cell density reached 10^8^ CFU/mL. *Lactobacillus plantarum* m506 (isolated from naturally fermented Chinese sauekraut) was inoculated into MRS medium to culture at 30 °C until the cell density reached 10^9^ CFU/mL. *Bacillus* sp. K102 was inoculated into Luria–Bertani (LB) broth to culture at 30 °C until the cell density reached 10^9^ CFU/mL. *Aspergillus oryzae* RIB40 [21] was inoculated into PDB medium (potato broth 200 g/L and glucose 20 g/L) to culture at 30 °C until the mycelia reached 0.1 g/mL. The kelp scraps were rinsed with sterile water three times to remove impurities, cut into approximately 1 cm × 2 cm pieces, and placed in a sterilizing pot for sterilization 15 min at 115 °C as the fermentation substrate. Next, 50 g of kelp scraps was inoculated with the above activated food-scale microbes in a 300 mL Erlenmeyer flask and fermented at 30 °C for 30 days. For natural fermentation, cleaned kelp scraps (100 g) and salt (20 g) were directly added into a 300 mL Erlenmeyer flask and cultured for 30 days at room temperature. The uninoculated and sterilized kelp scraps were used as the control.

### 2.3. 16s rRNA Sequencing

Approximately 1 g of kelp sauce was suspended in 10 mL of autoclaved distilled water. The resulting mixture was initially filtered through an eight-layer gauze to eliminate water-insoluble particles, followed by filtration through a 0.22 µm Millipore^®^ filter (Merck KGaA, Darmstadt, Germany) for sterilization and finer particulate removal. Total DNA extraction and purification were conducted using the TGuide S96 Magnetic Soil/Stool DNA Kit (TianGen Biotech, Beijing, China) following the manufacturer’s protocol. The extracted DNA from three samples underwent amplification targeting the V3–V4 hypervariable regions of the 16S rDNA gene using a primer pair (forward: CCTACGGGAGGCAGCAG; reverse: GGACTACHVGGGTWTCTAAT). Sequencing and quality control were carried out on Biomarker’s standardized platform (Beijing, China). The sequencing reads were clustered taxonomically using USEARCH v10.0, applying a 95% identity threshold and a minimum abundance of 0.005% [22]. Taxonomic prediction analyses were conducted using the classify–consensus–blast method in QIIME2 by querying the bacterial reads against the reference Greengenes (version 13.5) database [23] or classify-sklearn method. For statistical comparisons of bacterial diversity indices, QIIME2 (https://qiime2.org/ (accessed on 1 February 2024)) was used for the statistical analysis [24]. Analysis of α-diversity indices, including Shannon, Simpson, and chao-1 diversity indices based on OTU abundance, was performed using QIIME with the MOTHUR function.

### 2.4. Analysis of Polysaccharide Lyase and Protease Activities

The alginate lyase activity in the kelp sauce was assessed using ultraviolet absorption spectroscopy [25,26]. The assay was conducted by preparing a reaction mixture containing 0.5% (*w*/*v*) substrates (either standard sodium alginate, PloyG, or PloyG) in 50 mM phosphate buffer at pH 7.5. The mixture was incubated at 40 °C for 10 min to allow for the enzymatic reaction to proceed. To halt the reaction, the mixture was subsequently boiled for 10 min at 100 °C. Enzyme activity was quantified by measuring the increase in absorbance at 235 nm (OD_235_) with a SpectraMax iD3 Multi-Mode Microplate Reader (Molecular Devices, CA, USA). One unit of lyase activity (U) was defined as the amount of enzyme required to produce an increase of 0.1 OD_235_ units per minute under the specified conditions.

The protease activity was assessed using the Folin phenol reagent method with casein as the substrate [27]. The reaction mixture comprised 100 μL of diluted supernatant from the kelp sauce and 100 μL of 1% (*w*/*v*) casein solution in 10 mM phosphate buffer (pH 3.0), which was incubated at 30 °C for 10 min to facilitate enzymatic hydrolysis. The reaction was terminated by adding 200 μL of 0.4 M trichloroacetic acid. The mixture was then centrifuged at 12,000× *g* for 3 min. Next, 100 μL of the resulting supernatant was combined with 500 μL of 0.4 M sodium carbonate (Na_2_CO_3_) and 100 μL of the Folin phenol reagent (Sangon Biotech, Shanghai, China). This mixture was incubated at 50 °C for 20 min, after which absorbance was measured at 650 nm using a spectrophotometer. The inactivated kelp sauce supernatant was used as the control. One unit (U) of protease activity was defined as the number of enzymes required to hydrolyze casein and release 1 μg of tyrosine per minute.

### 2.5. Isolation and Purification of Umami Peptides

For pre-isolation, 5 g of kelp sauce was dispersed in 25 mL of ultrapure water and subsequently filtered through eight layers of gauze to eliminate insoluble kelp residues. Then, the filtrate was centrifuged at 12,000× *g* for 10 min to remove any remaining insoluble particles. To enrich the peptide mixture, the clarified solution was subjected to precipitation with 80% food-grade ethanol at 4 °C. The precipitated peptides were collected and subsequently freeze-dried for further analysis.

According to reports, umami peptides, as small-molecule peptides, typically have a molecular weight below 3 kDa [28]. The lyophilized powder was re-dissolved in ultrapure water and ultrafiltered through a 3 kDa molecular weight cutoff membrane (Millipore, Darmstadt, Germany) to eliminate components with molecular weights greater than 3 kDa. The filtrate, now enriched in low-molecular-weight (≤3 kDa) peptides, was collected and freeze-dried. The lyophilized powder was once again dissolved in ultrapure water and filtered through a 0.45 μm hydrophilic membrane (Millipore, Germany) to ensure clarity. Subsequently, the filtrate was fractionated using Sephadex G-15 gel filtration chromatography at a flow rate of 1.0 mL/min at 25 °C, with ultrapure water as the eluent. This process yielded four distinct peptide fractions, one of which exhibited umami taste characteristics and was selected for subsequent peptide sequence identification.

### 2.6. UPLC–MS

The fraction exhibiting the most pronounced umami taste was analyzed using high-performance liquid chromatography (HPLC) on a Thermo Scientific Ultra Performance LC system (Thermo Scientific, Waltham, MA, USA) as described in Ref. [29], with modification. The peptide sample was separated on a C18 column (75 µm * 150 mm, 3 µm, Dr. Maisch GmbH (Dr. Maisch, Ammerbuch, Germany)) maintained at a temperature of 40 °C with a flow rate of 200 nL/min. The mobile phases consisted of the following: mobile phase A: 0.1% formic acid in water; mobile phase B: 0.1% formic acid in acetonitrile (70%); and water (30%). The elution gradient was programmed as follows: 0–2 min, 5% B; 2–10 min, 5–10% B; 10–20 min, 10–15% B; 20–30 min, 20–30% B; 30–45 min, 40% B; 45–53 min, 40–100% B. Detection of eluting peaks was performed at a wavelength of 220 nm.

Peptides were identified using a Thermo Scientific Q Exactive HF-X hybrid quadrupole–Orbitrap mass spectrometer (Thermo Scientific, MA, USA). The MS was operated in Data Dependent Acquisition (DDA) mode, recording spectra in positive ion mode over a parent ion (*m*/*z*) range of 350 to 1800. The instrument settings were as follows: spray voltage was 2.2 kV, and the capillary temperature was 270 °C. The primary mass spectrum scan mode was 350–1800 *m*/*z*, MS resolution was 60,000, and separation width was 3.00 Da. Ten ions with the highest ionic strength in the primary mass spectrometry were selected for MS–MS spectrometric dissociation. All raw MS–MS data files were processed with MaxQuant 2.4.14.0 (Thermo Scientific, MA, USA) for peptide identification and quantification analysis.

### 2.7. Taste Analysis of Kelp Sauce and Umami Peptides via Electronic Tongue

The taste characteristics of kelp sauce and peptide solutions were measured at room temperature by the electronic tongue (Shanghai Bosin Industrial Development Co., Ltd., Shanghai, China) equipped with six transducers (Platinum, Aurum, Palladium, Tungsten, Titanium, and Argentum), as previously reported [30]. The electronic tongue was preheated for 30 min before analysis. Before and after the test, the transducer probes were washed with ultrapure water three times. Each sample was measured five times. Five standard solutions (5 points), including citric acid (0.1%, *w*/*v*), sucrose (1%, *w*/*v*), MSG (0.5%, *w*/*v*), L-isoleucine (0.5%, *w*/*v*), and NaCl (0.5%, *w*/*v*) solutions, were employed to calibrate sour, sweet, umami, bitter, and salty tastes, respectively.

### 2.8. Molecular Docking of Umami Peptides and Receptors TAS1R1–TAS1R3

As the ligand, umami peptides were drawn by Pymol 3.1 and energy-minimized by Discovery Studio 2019 (San Diego, CA, USA). Homology modeling of the umami receptor TAS1R1–TAS1R3 complex was constructed using the SWISS-MODEL (https://swissmodel.expasy.org/interactive (accessed on 1 February 2024)), and its quality was assessed via the Ramachandran plot residual percentage. The amino acid sequences for TAS1R1 (accession number Q7RTX1) and TAS1R3 (accession number Q7RTX0) were retrieved from UniProt (https://www.uniprot.org (accessed on 1 February 2024)). For homology modeling, the metabotropic glutamate receptor structure (PDB ID: 1EWK) served as the template [31]. Then, the preliminary homology model was imported into Discovery Studio and the model was optimized using the minimization protocol. The coordinates for the active center of T1R1 and T1R3 are as follows: x = 17.5291, y = −3.99985, and z = −16.9513; x = 23.9686, y = −3.7666, and z = −12.6583. To ensure comprehensive interaction analysis between the umami peptides and the receptor, the radius of the docked active sphere was expanded to 19 Å.

### 2.9. Statistical Analysis

SPSS software was used for statistical analysis (version 19.0, SPSS Inc., Chicago, IL, USA). All data are presented as the means ± standard deviations (SDs). The radar chart of data from electronic tongue analysis was generated via Origin 2024 (Origin Lab Corporation, Northampton, MA, USA). Significant differences were determined by using one-way ANOVA analysis, with *p* ≤ 0.05 considered statistically significant.

## 3. Results

### 3.1. Fermentation of Kelp Scraps into Kelp Sauces by Common Microbes

The seaweed *Laminaria japonica* is consumed as a common dietary food that has nourished billions of people. However, some consumers are deterred from directly consuming fresh kelp due to its strong fishy odor. In the food processing industry, the thin edges and roots of kelp, which have a less desirable taste, are often discarded. Moreover, kelp is predominantly composed of polysaccharides and proteins that are difficult to digest, impacting the absorption of its nutrients. Microbial fermentation can address these challenges by not only converting and eliminating undesirable chemical components but also breaking down complex biomacromolecules into smaller, more easily absorbed molecules. Additionally, this process facilitates the production of new small-molecule flavor compounds. To recycle kelp biomass, this study aimed to transform kelp by-products into sauces through microbial fermentation. Initially, we attempted to ferment kelp materials using common food-grade microorganisms, including *L. plantarum*, *Bacillus* sp., *Saccharomyces cerevisiae*, *Pichia pastoris*, and *Aspergillus oryzae* (Table 1). According to the comparison of OD_600_ value increases, when cultivated in a kelp medium, the two yeast strains, *S. cerevisiae* and *P. pastoris*, exhibited the fastest growth, followed by *Bacillus* species, while *L. plantarum* demonstrated the slowest growth rate. The growth rate of *A. oryzae* showed a comparable increase to pure cultures, indicating that kelp does not exert inhibitory effects on microbial growth. Further observations revealed that these microorganisms caused no destructive effects on the physical structure of kelp. Even after 30 days of fermentation, neither individual strains nor combinations of these microorganisms could effectively degrade the kelp tissue.

### 3.2. Natural Fermentation of Kelp Scraps into Kelp Sauces by Endotrophic or Epiphytic Microbiome

**Figure 1:**
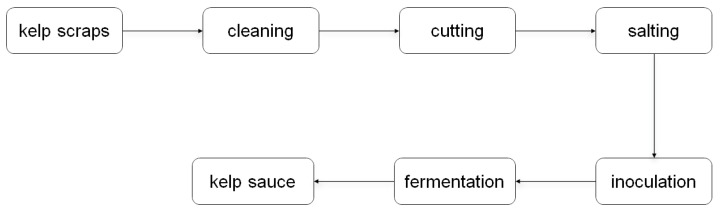
The roadmap of preparation of kelp sauce by microbial natural fermentation.

**Figure 2:**
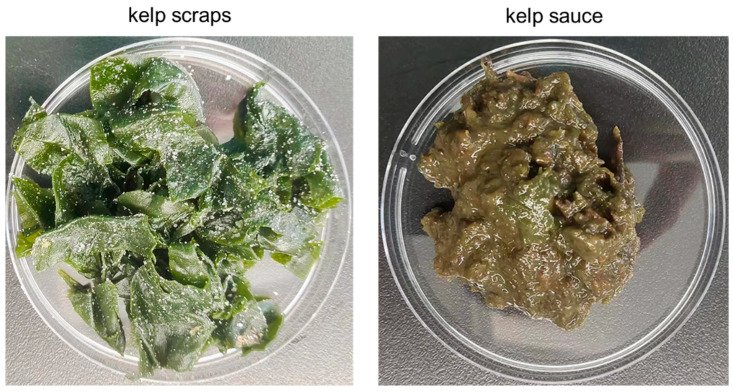
Fermentation of kelp scraps into kelp sauce by natural microbial flora.

It was widely reported that brown macroalgal endophytic and epiphytic microbial communities possess a rich diversity of carbohydrate-active enzymes, including alginate lyases [28]. Therefore, we tried to carry out a natural fermentation approach using endotrophic or epiphytic microbial strains associated with kelp (Figure 1). Fresh kelp scraps from three aquiculture areas (ND, HD, KD) were selected as raw materials, bypassing high-temperature processing to preserve their associated microorganisms. These kelp samples were then salted with 20% (*w*/*v*) food-grade sodium chloride. The solid fermentation was carried out in food-grade packaging boxes under dark conditions at temperatures ranging from 15 to 25 °C. After 30 days of fermentation, significant changes in morphology, texture, color, and luster were notably observed in the kelp tissue from the HD sample (Figure 2). The blade-like structure of the kelp had decomposed into a paste-like sauce, and its color transitioned from bright green to light brown. In addition, the undesirable fishy odor of the seaweed was entirely eliminated, giving way to a pronounced umami taste following microbial fermentation.

### 3.3. Sequencing Analysis of Microbiome of Kelp Sauces

To investigate the details of the active microbiome, metabolite composition, and changes in the morphology and appearance of kelp during the natural fermentation, we divided the fermentation process of kelp sauce into three distinct stages. The first stage was 8 days after inoculation (HD1), when the light green color of the kelp begins to fade. The key feature of stage 2 (15 days after inoculation) was that a part of the kelp pieces started to disintegrate into a sauce-like consistency, with the green color fading away. At stage 3 (30 days after inoculation), all kelp scraps were fully degraded into a homogeneous kelp sauce, with no visible pieces of kelp remaining. This marks the completion of the fermentation process.

**Figure 3:**
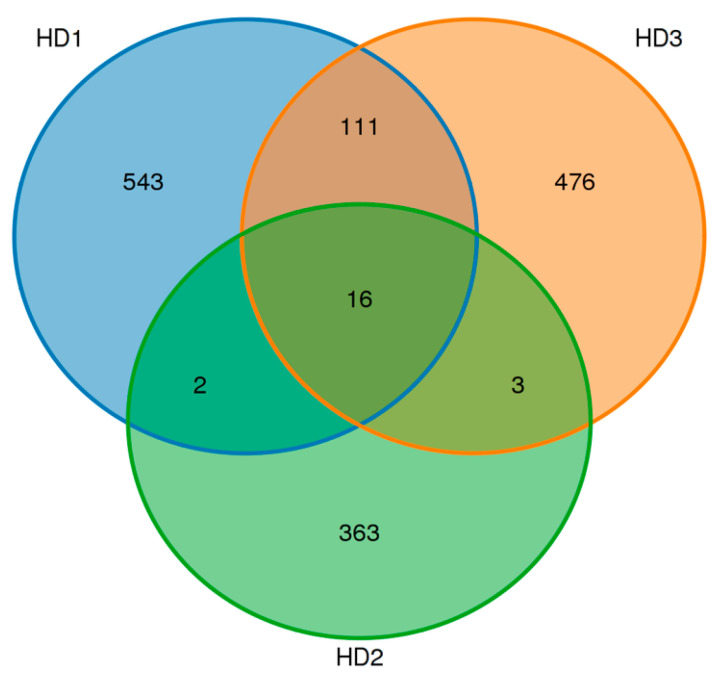
Venn diagram of OUTs from three different fermentations of kelp sauces.

To explore the functional and active microbiota involved in kelp sauce fermentation, samples from three stages (HD1, HD2, and HD3) were collected and analyzed using 16S rRNA high-throughput sequencing. After applying quality control measures, including filtering for low-quality and length criteria, we obtained a total of 70,881, 73,629, and 71,878 high-quality 16S rDNA sequences for HD1, HD2, and HD3, respectively. OUT analysis revealed 672, 384, and 606 OTUs for HD1, HD2, and HD3, respectively. The number of OTUs exhibited a dynamic pattern, initially increasing and then gradually decreasing. Notably, 16 OTUs were shared across all three samples, indicating their central role in the kelp fermentation process (Figure 3).

**Figure 4:**
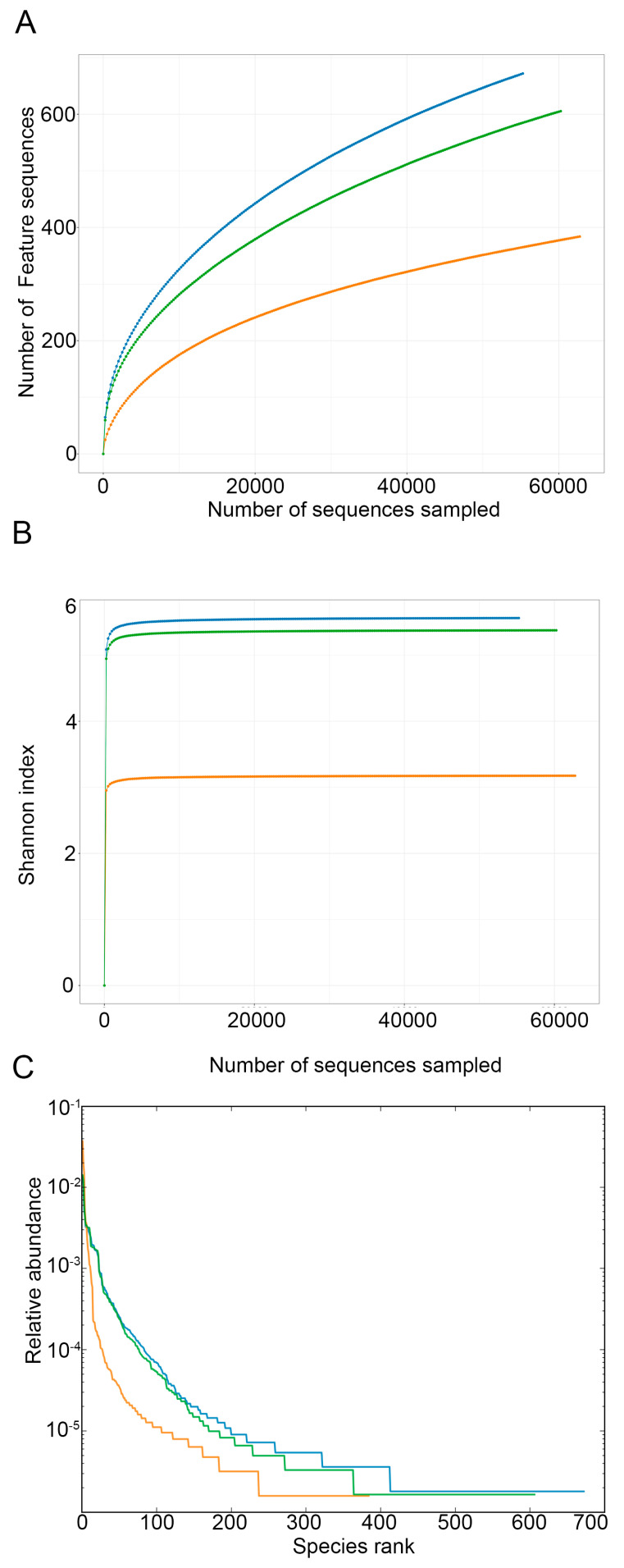
Bacterial diversity and abundance analysis of kelp sauces at different stages. Blue: HD1; orange: HD3; green: HD2. (**A**) Rarefaction curve (**B**) Rarefaction curve (**C**) Rank Abundance Curve.

The bacterial diversity and species abundance across different fermentation stages were evaluated using Rarefaction, Shannon index, and rank abundance analyses. Rarefaction analysis revealed that HD2 exhibited a more stable curve compared to the other two stages, indicating a well-established and diverse bacterial community in this sample (Figure 4A). For Alpha diversity, Shannon index values of different samples were calculated, and the results indicated that HD1 and HD3 had higher bacterial diversity than HD2 (Figure 4B). An analysis of the rank abundance curves of different samples revealed that HD1 harbored the largest amount of OTUs, followed by HD3, while HD2 displayed a contracted bacterial community with fewer OTUs (Figure 4C).

**Figure 5:**
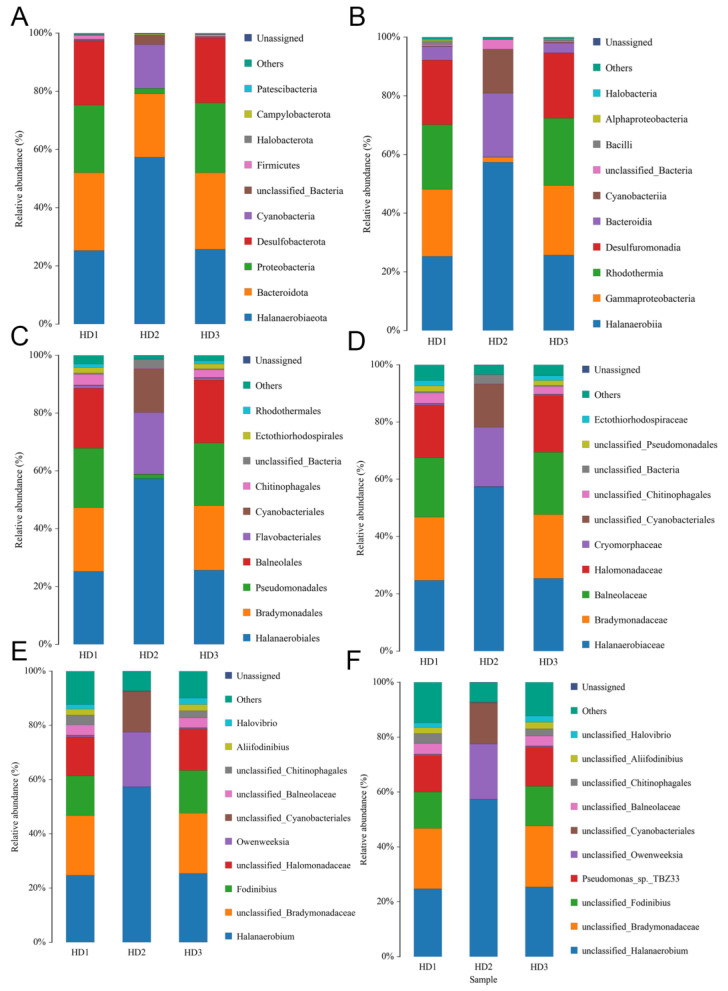
Bacterial community analysis of kelp sauces. (**A**) Species distribution at the phylum level (**B**) Species distribution at the class level (**C**) Species distribution at the order level (**D**) Species distribution at the family level (**E**) Species distribution at the genus level (**F**) Species distribution at the species level.

Compared to HD2, both HD1 and HD3 exhibited significantly higher bacterial diversity at all taxonomic levels, from phylum down to species (Figure 5A–F). An analysis of the relative abundance of bacterial phyla throughout the entire kelp fermentation process identified three dominant phyla, including *Halanaerobiaeota*, *Bacteroidota*, and *Proteobacteria* (Figure 5A). *Halanaerobiaeota* predominated in the early stage of fermentation, accounting for 25.3% at HD1 and increasing to 57.4% at HD2 before sharply declining to 25.8% at HD3 (Figure 5A). Interestingly, *Desulfobacterota* displayed high abundance in the early and late stages (22%) but was greatly reduced during mid-fermentation (Figure 5A). At the genus level, *Halanaerobium* was identified as the predominant genus during the fermentation, with its relative abundance increasing from 24.8% at HD1 to 57.4% at HD2, then decreasing to 25.3% at HD3 (Figure 5E). Intriguingly, unclassified genera within the family Halomonadaceae were specifically identified in HD1 (14.4%) and HD3 (15.3%) but were absent in HD2 (Figure 5E).

### 3.4. Analysis of Active Enzymes During Fermentation of Kelp Sauces

**Figure 6:**
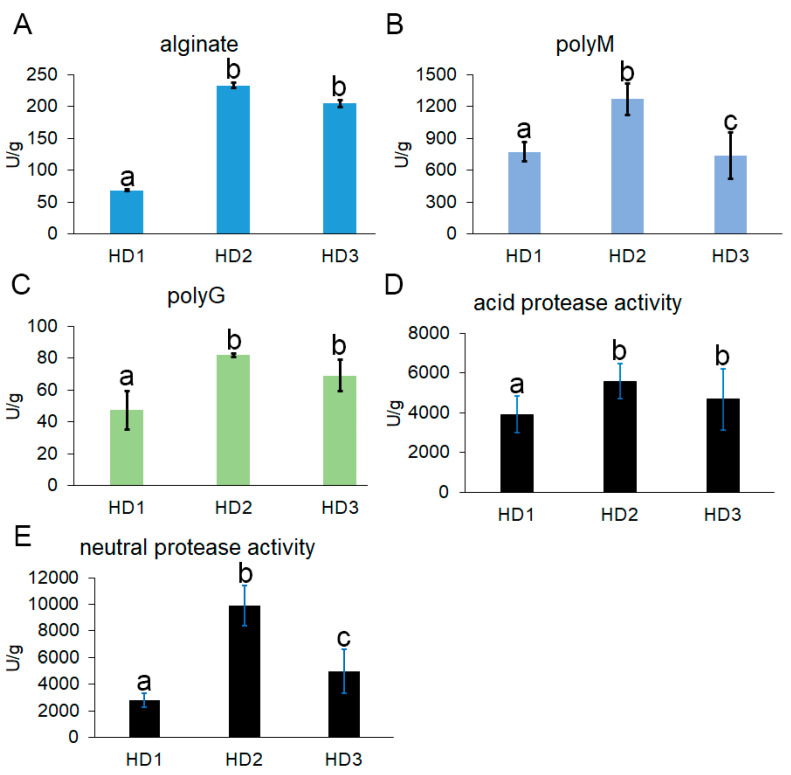
Activity levels of active enzymatic during the fermentation process. Different letters indicate significant differences, *p* ≤ 0.05. (**A**) alginate lyase activity with alginate as the substrate (**B**) alginate lyase activity with polyM as the substrate (**C**) alginate lyase activity with polyG as the substrate neutral protease activity (**D**) acid protease activity (**E**) neutral protease activity.

The tissue of kelp comprises high-molecular-weight alginate polysaccharides and proteins. During the fermentation process to produce kelp sauces, microorganisms generate specific enzymes that depolymerize alginate into low-molecular-weight oligosaccharides and hydrolyze proteins into peptides or amino acids. Therefore, enzymes play a crucial role in kelp fermentation. Investigating the enzyme activity during kelp fermentation, the activities of alginate lyase and protease at various stages of the fermentation process were determined. We employed three substances (alginate, polyguluronate polyM, and ployG) to measure alginate lyase activity, as well as casein for protease activity. Overall, both alginate lyase and protease activities initially increased, peaking at the HD2 stage, before gradually decreasing throughout the entire fermentation process (Figure 6). This trend mirrored the changes observed in bacterial diversity. Interestingly, this alginate lyase in the fermentation broth exhibited a significant preference for polymannuronate (PolyM) over other substrates. When PolyM was used as the substrate, the highest enzyme activity reached 1267 U/g, several times higher than when using alginate (233 U/g) or polyG (82 U/g) as substrates (Figure 6A–C). When alginate was used as the substrate, the highest enzyme activity was observed in HD2, reaching 233 U/g, and then gradually decreased to 204 U/g (Figure 6A). When polyM was used as the substance, the alginate lyase activity increased from 771 U/g at HD1 to a peak of 1267 U/g at HD2, before declining to 733 U/mL at HD3 (Figure 6B). For polyG, the activity also increased and peaked at HD2 but remained stable thereafter until the end of fermentation (Figure 6C). An analysis of protein-hydrolyzing enzymes revealed that neutral protease activity was the highest in this fermentation broth, reaching up to 9916 U/g, followed by acid activity, while alkaline protease activity was nearly undetectable (Figure 6D,E). Similarly, protease activity also displayed an initial increase followed by a gradual decrease or stabilization. The level of acid protease increased from 3924 U/g at HD1 to 5591 U/g at HD2 and remained stable (Figure 6D). The level of neutral protease activity started at a low level of 2788 U/g, reached its peak at HD2, and subsequently declined to 4993 U/g towards the late stage of fermentation (Figure 6E).

### 3.5. Taste Analysis of Kelp Sauce

**Figure 7:**
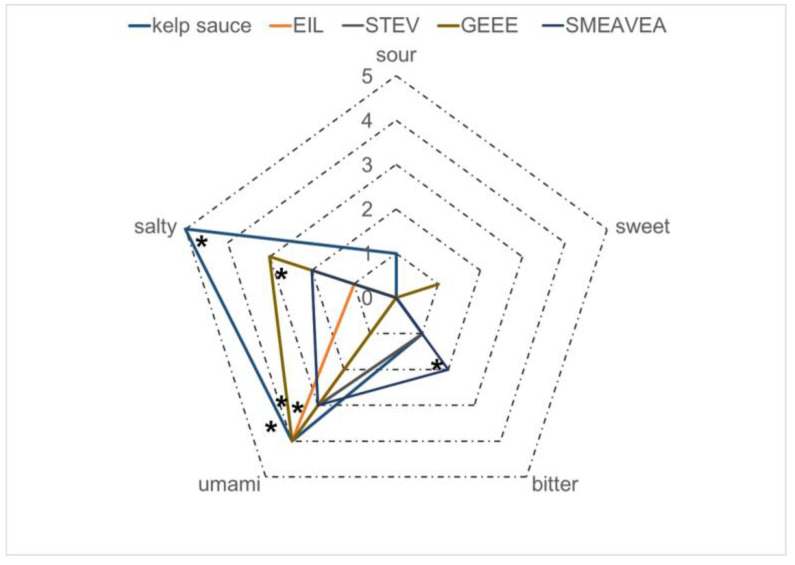
Electronic tongue analysis of the taste of kelp sauces and umami peptide. * *p* ≤ 0.05 considered statistically significant.

Electronic tongue analysis was conducted on kelp sauces to assess their umami taste and other sensory characteristics. For unbiased sampling, kelp sauces that had undergone 30 d of natural fermentation (HD3) were selected for taste evaluation. Our electronic tongue analysis results showed that the kelp sauce exhibited sensations of saltiness, umami, sourness, bitterness, and a slight fishy note, and saltiness and umami were the most prominent flavors (Figure 7). In comparison, the umami intensity of the uninoculated and sterilized kelp scraps is low and emits a distinct fishy odor.

We speculate that the saltiness in kelp sauce primarily stems from the added salt, as well as amino acids and peptides generated through hydrolysis. Kelp peptides are also significant contributors to both the saltiness and umami taste of kelp sauce. Subsequently, we employed LC–MS (liquid chromatography–mass spectrometry) to analyze the peptide components in kelp sauce. Given that desalted HD3 exhibits a stronger umami flavor compared to HD1 and HD2, we selected HD3 for peptide analysis (Figure S1). After step-wise purification, one component with umami sensation was used for peptide identification. A total of 16 peptides were identified in the component of HD3 kelp sauce (Table 2), and the top four peptides with the highest abundance were chemically synthesized for further investigation (Figures S2–S5). Electronic tongue analysis confirmed that all four peptides displayed umami taste. Among them, GEEE exhibited the strongest umami sensation, accompanied by some degree of saltiness. The peptide EIL also has a strong umami taste without saltiness. STEV and SMEAVEA showed moderate umami tastes, with SMEAVEA additionally exhibiting a slight bitterness. Glutamic acid is a common feature among these four peptides, suggesting its importance in conferring umami taste. This insight could be valuable for identifying umami peptides in other foods.

### 3.6. Molecular Docking of Umami Peptides and Receptors

Recent advances have uncovered the molecular mechanisms underlying taste perception and recognition. Receptors involved in umami taste perception in human taste bud cells include T1R1–T1R3, mGluR4, and mGluR1 [18]. To elucidate the interaction between umami peptides and their receptors, we performed molecular docking studies using Autodock 4.2.6 software. In this study, we first predicted the three-dimensional structures of two key umami receptors, T1R1 and T1R3, using the AlphaFold2 method via the online SWISS-MODEL platform (Figure S6). The predicted models of T1R1–T1R3 were evaluated using a structure assessment web server, yielding a high MolProbity score of 0.90 for T1R1 and 0.98 for T1R3. The Ramachandran favored region showed that 97.50% and 96.59% of the amino acid residues were located in adored regions for T1R1 and T1R3, respectively (Figure S6), indicating that these models are highly reliable. Then, molecular docking was conducted between the umami receptor T1R1–T1R3 and two umami peptides derived from kelp sauce to investigate the sense mechanism of these peptides. The results demonstrated that both peptides could form a stable complex with the T1R1–T1R3 receptor. The interactions contributing to establish the umami peptides and the T1R1–T1R3 receptor complex include conventional hydrogen bonds, van der Waals interactions, carbon–hydrogen bonds, and alkyl and π–alkyl interactions.

**Figure 8:**
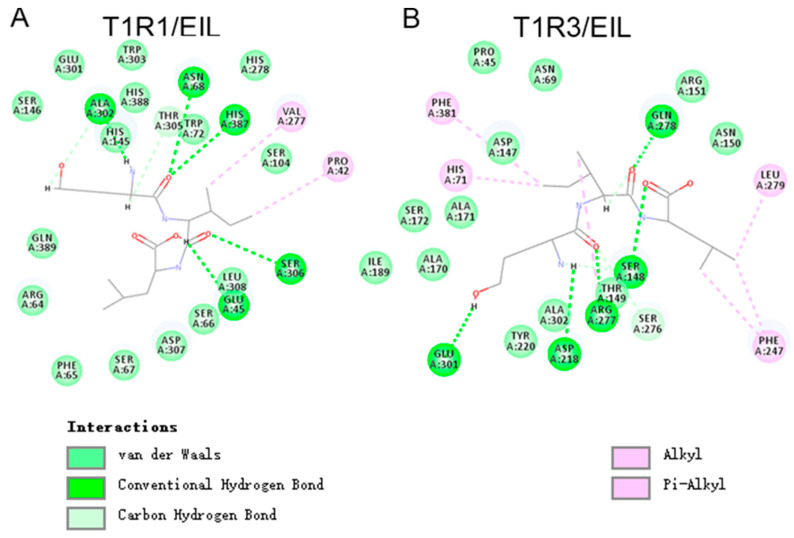
The molecular docking models of the peptides EIL with T1R1 and T1R3. (**A**) EIL and T1R1 interaction (**B**) EIL and T1R3 interaction.

A detailed analysis of the interactions at the active sites of the T1R1–T1R3 receptors is presented below. Among the four peptides, GEEE and EIL exhibited significantly lower docking interaction energy values (57.2407 and 47.0961, respectively) compared to the other two peptides (78.2027 for GEEE and 82.8245 for SMEAVEA), suggesting that these two peptides form a tighter and more stable complex with the receptors than the others. As shown in Figure 8A,B, the ligand umami peptide EIL mainly interacts with T1R1–T1R3 receptor residues through van der Waals and conventional hydrogen bonding. EIL formed five conventional hydrogen bonds with residues Glu45, Asn68, Ala302, Ser306, and His387 in T1R1, as well as Ser148, Asp218, Arg277, Gln278, and Glu301 in T1R3. EIL form van der Waals interactions with 13 specific sites on T1R1 (Arg64, Phe65, Ser66, Ser67, Trp72, Ser104, Ser146, His278, Glu301, Trp303, Leu308, His388, and Gln389) and 11 sites on T1R3 (Pro45, Asn69, Asp147, Asn150, Arg151, Ala170, Ala171, Ser172, Ile189, Ayr220, and Ala302). Furthermore, the peptide EIL establishes interactions via alkyl bonds, contributing to its stable association with the receptors.

**Figure 9:**
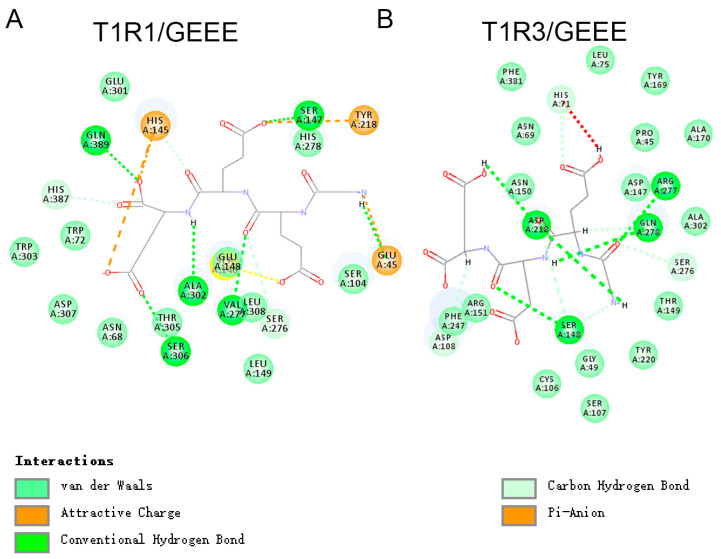
The molecular docking models of the peptides GEEE with T1R1 and T1R3. (**A**) GEEE and T1R1 interaction (**B**) GEEE and T1R3 interaction.

The ligand umami peptide GEEE mainly interacts with the T1R1–T1R3 receptor residues through multiple forces, including van der Waals interactions, conventional hydrogen bonds, attractive charge–charge interactions, carbon–hydrogen bonds, and Pi–anion forces (Figure 9). Interestingly, three glutamic acids of GEEE formed four conventional hydrogen bonds with Ser147, Val277, Ser306, and Gln389 of the T1R1 receptor (Figure 9A). Four conventional hydrogen bonds were observed between GEEE and T1R3, involving residues Ser148, Asp218, Arg277, and Gln278. Additionally, van der Waals interactions were detected at over ten sites of T1R1 and T1R3. Interestingly, the peptide formed three attractive charge bonds with T1R1, with sites including Glu45, His145, and Tyr218, which was the first report that the charge interaction may contribute to enhancing the interaction between the umami peptides and receptors. In addition, the major active docking sites of the two umami peptides include residues such as Ser306, Ser148, Asp218, Arg277, and Gln278, which are present in the docking interfaces of all three umami peptides, and these partial sites have also been previously demonstrated.

In summary, the interaction between umami peptides and the T1R1–T1R3 taste receptor is mediated by a combination of hydrogen bonding, van der Waals forces, and electrostatic interactions. Key residues from T1R1–T1R3, including Gln, Ser, Arg, and Asp, play a crucial role in binding the three umami peptides. Basic residues, such as His and Tyr, contribute to attractive charge–charge interactions between the glutamic acid of peptides and basic amino acids.

## 4. Discussions

Kelp (*L. japonica*) has become the world’s most produced edible brown algae, with China being the largest cultivator [32]. As one kind of nutritious food, kelp is primarily consumed in the form of fresh vegetables in most Asian countries [33]. The thinner edges and roots of kelp, which are discarded due to poor texture, account for nearly 40% of the kelp biomass. These by-products are rich in proteins, polysaccharides, minerals, vitamins, trace elements, and other nutrients [34]. Therefore, there is an urgent need to develop new food-processing technologies to utilize these discarded components. Microbial fermentation is a common and efficient method that is used to preserve food and improve sensory quality [35]. Our results showed that common microbes (individually or in combination) for food fermentation could not decompose the kelp. It has been reported that kelp in a raw state could not support the growth of *L. plantarum*, and that only the combination of *L. plantarum* and natural microbiota can ferment kelp into sauerkraut-style food [36,37]. Fermentation with *L. plantarum* and *S. cerevisiae* is an efficient strategy to remove the fishy malodor compounds, but it did not change the texture, organizational structure, or nutritional ingredients [38,39]. We hypothesized that this might be because kelp cell walls are primarily composed of alginate, an algal polysaccharide, and the selected microorganisms lack the necessary alginate lyase enzymes to break down the cell wall. It was reported that brown macroalgal (including kelp) endophytic and epiphytic microbial communities possess a rich diversity of carbohydrate-active enzymes, including alginate lyases [31].

Actually, the kelp products were completely fermented into sauce-like foods by a native microbial community, which was not reported previously. We believe that these findings hold three key implications: Firstly, the microbiome contains novel microbial resources, with the potential to discover a large number of new species or even genera, particularly halotolerant microorganisms. Further investigation, using either culture-dependent or culture-independent methods, will be required to identify these novel genera. Halanaerobium has widespread use in fish sauce fermentation due to their ability of the secretion production of halophilic protease, as well as esterase/lipase under high concentrations of salt [40,41,42,43]. Secondly, our results indicate that a wide range of biomacromolecule-degrading enzymes participate in the fermentation process. These salt-tolerant novel enzymes also warrant further exploration and research. Lastly, but crucially, kelp paste post-fermentation contains small-molecule oligosaccharides and oligopeptides. The structural characteristics and biological activities of these bioactive molecules deserve in-depth investigation.

Natural umami or umami-enhancing peptides are increasingly flavored by consumers due to their ability to enhance food flavor, reduce sodium intake, and improve appetite [18]. Until now, most umami peptides have been isolated from animals. For example, between 2018 and 2022, a total of 159 umami peptides were identified, among which 117 were isolated from animals and 42 were plant-derived [18]. Our findings suggested that seaweed and its fermented foods are the emerging source of umami peptides, which are further supported by amino acid composition. It has been revealed that seaweed proteins are rich in umami amino acids, with glutamic acid and aspartic acid being the most abundant [44].

## 5. Conclusions

In this study, we transformed kelp sweepings into an umami peptide-enriched kelp sauce through natural microbial fermentation rather than single food-grade microorganisms, providing a novel approach for the high-value utilization of kelp processing by-products. Our results demonstrate that halotolerant microorganisms and their diverse enzymes play critical roles during kelp paste fermentation, establishing a valuable resource repository for the screening and isolation of functional microorganisms and the discovery of novel enzymes. To investigate the umami components, four umami peptides were identified, proving that seaweeds and their fermented products serve as significant sources of food-derived umami substances. Molecular docking analysis demonstrated that the interaction between these umami peptides and the T1R1–T1R3 taste receptor was mediated by a combination of hydrogen bonding, van der Waals forces, and attractive charge interactions. The key residues involved in hydrogen bonding with the receptors included Ser306, Ser148, Asp218, Arg277, and Gln278. In conclusion, our study provides a novel pathway for converting low-cost kelp by-products into value-added functional foods, thereby offering new opportunities for sustainable food processing and flavor enhancement.

## Figures and Tables

**Table 1:** Fermentation of kelp scraps by microbes.

Strains	Substance	Fermentation Temperature
*L. plantarum* m506	sterilized kelp scraps	30
*S. cerevisiae* mj1003	sterilized kelp scraps	30
*P. pastoris* X33	sterilized kelp scraps	30
*Bacillus* sp. K102	sterilized kelp scraps	30
*A* *. oryzae RIB40*	sterilized kelp scraps	30
*S. cerevisiae* mj1003/*L. plantarum* m506	sterilized kelp scraps	30
*A. oryzae RIB40*/*L. plantarum* m506	sterilized kelp scraps	30
Natural microbial flora	cleaned kelp scraps	RT

**Table 2:** Sixteen umami peptides were identified in the kelp sauce.

No.	Sequence	Length	Mass	Peptide No.	Score
1	SMEAVEA	7	735.1	21	150.0
2	IVSLAPEVL	9	939.6	10	148.2
3	EIL	3	373.5	18	89.8
4	ISFLNK	6	720.4	12	87.8
5	SVEEIK	6	703.4	11	84.0
6	LENAIR	6	714.4	14	69.1
7	GEEE	4	462.4	26	63.2
8	IDHIYRDR	8	1086.6	5	9.4
9	RYQMGYIK	8	1057.5	9	9.2
10	STEV	4	434.4	17	5.9
11	EIPPATYNFRLYSGKMLELYGR	22	2617.3	3	2.8
12	SRVWKYQMGQMPISQLSK	18	2166.1	2	1.0
13	YKKDHDDDDPVDILVDVDFDK	21	2505.2	3	0.5
14	DRFCFCAEALYKAQAETGEIK	21	2506.2	2	0.5
15	FFRSKLNICEQCGYHLK	17	2199.1	2	0.2
16	IWICNWNKNDNAT	13	1647.7	2	0.2

## Data Availability

The original contributions presented in the study are included in the article/Supplementary Material, further inquiries can be directed to the corresponding author.

## Supplementary Materials

The following supporting information can be downloaded at: https://www.mdpi.com/article/10.3390/foods14101751/s1, Figure S1: HPLC-MS analysis of peptides in HD3; Figure S2: MS analysis of the peptide EIL; Figure S3: MS analysis of the peptide GEEE; Figure S4: MS analysis of the peptide STEV; Figure S5: MS analysis of the peptide SMEAVEA; Figure S6: Ramachandran analysis of protein structure.

## Abbreviations

The following abbreviations are used in this manuscript:

FAO	Food and Agricultural Organization
ACE-1	Angiotensin-1-Converting Enzyme 1
MSG	Monosodium glutamate
LC–MS	Liquid chromatography–mass spectrometry
PolyM	Polymannuronic acid
PloyG	Polyguluronic acid
UPLC–MS	Ultra-performance liquid chromatography–mass spectrometry
SDs	Standard deviations
OTUs	Operational taxonomic units
