# Supplementary material for: Fermentation Preparation of Umami Sauce and Peptides from Kelp Scraps by Natural Microbial Flora
Source: Foods. 2025 May 15;14(10):1751. doi: 10.3390/foods14101751 (PMC12111377; doi:10.3390/foods14101751)

Fermentation Preparation of Umami Sauce and Peptides from Kelp Scraps by Natural Microbial Flora

Jizi Huang [1,2,†], Ruimei Wu [3,†], Yijing Wu [1], Feiyang Liang [1], Yiming Chen [1], Fujia Yang [1], Huawei Zheng [1], Zonghua Wang [1], Huibin Xu [1,*], Songbiao Chen [1] and Guangshan Yao [1,4,*]

Figure S1

HPLC-MS analysis of peptides in HD3

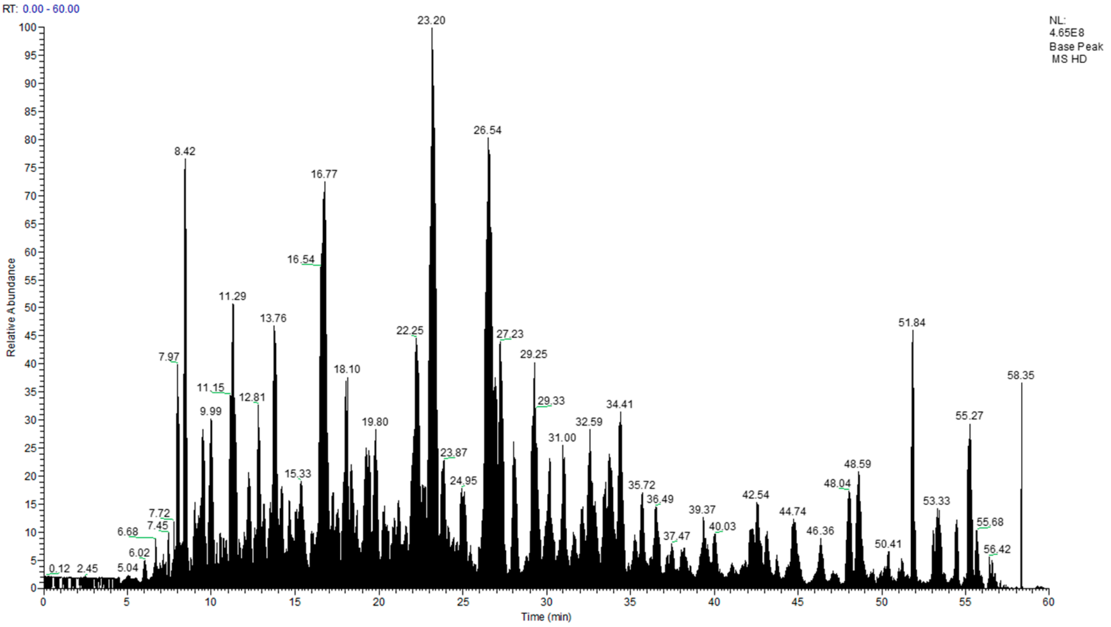

Figure S2

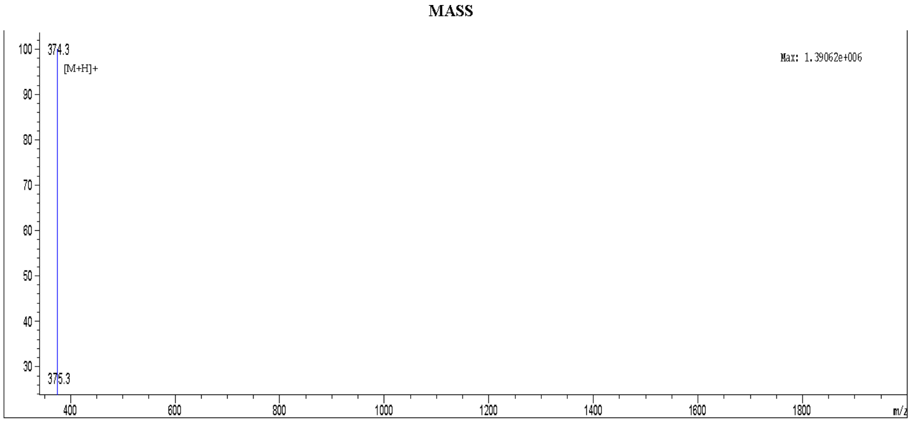

Figure S3

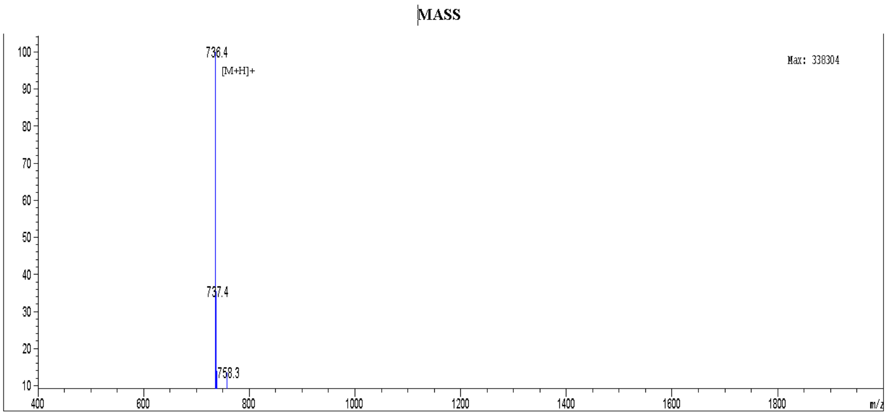

Figure S4

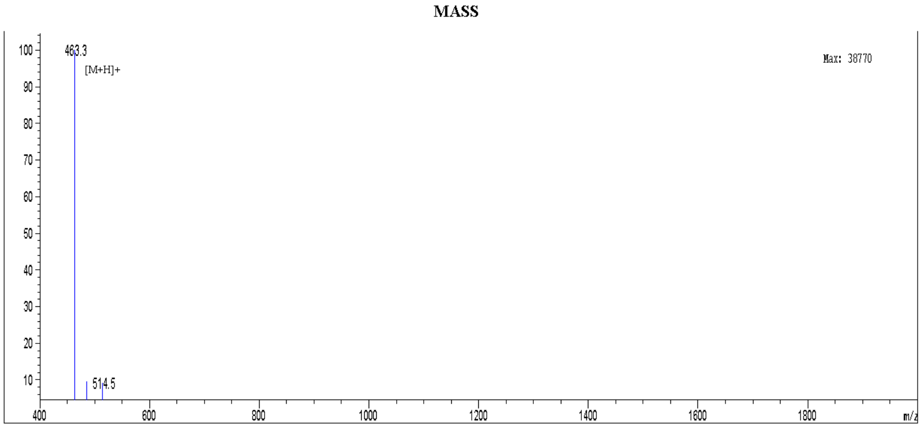

Figure S5

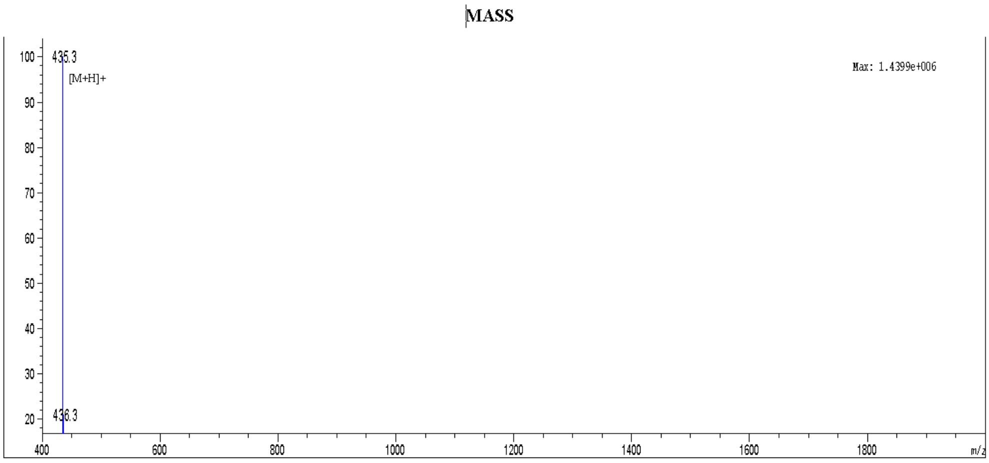

Figure S6

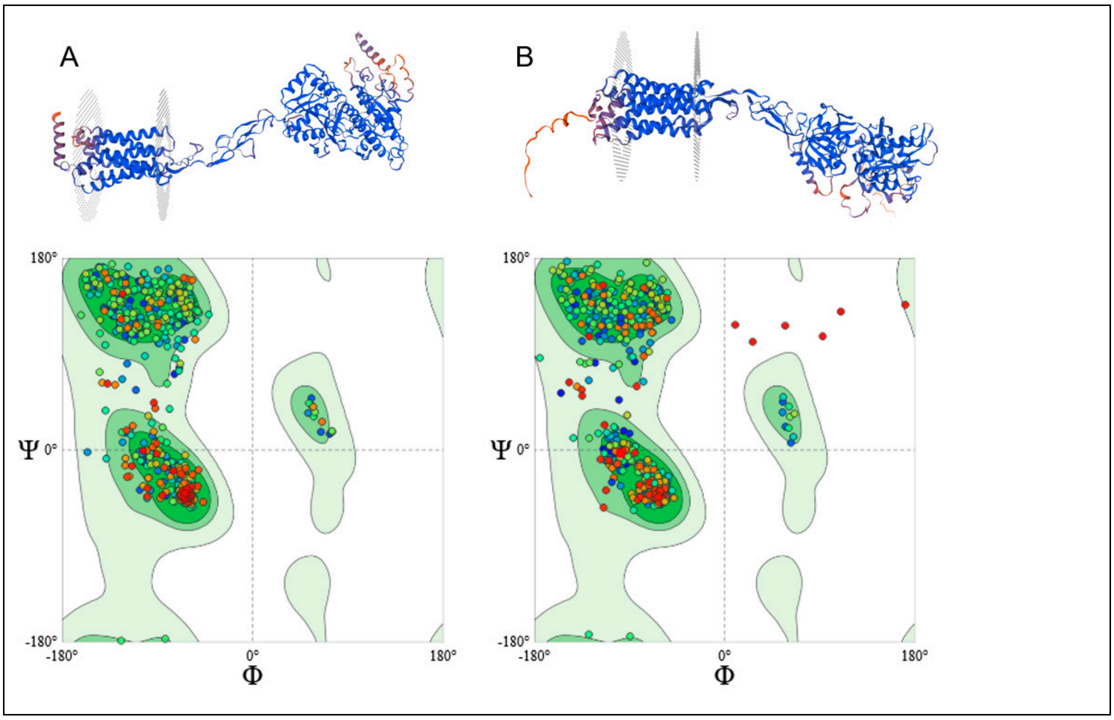

Supplement: Supplementary file 1 [file foods-14-01751-s001.zip › foods-3583908-supplementary.pdf]
